# Synergistic effects of gamma irradiation/salmide®, a sodium chlorite-based oxy-halogen, on microbiological control and the shelf life of chicken breasts

**DOI:** 10.1007/s11274-024-04183-9

**Published:** 2024-11-22

**Authors:** Reham M. M. Abdelkader, Assem Abolmaaty, Dina H. Amin

**Affiliations:** 1 Radiation Microbiology Department, National Center for Radiation Research and Technology, Egyptian Atomic Energy Authority, Cairo, Egypt; 2 Department of Food Science, Faculty of Agriculture, Ain Shams University, Cairo, Egypt; 3 Department of Microbiology, Faculty of Science, Ain Shams University, Abbasyia, Cairo, Egypt

**Keywords:** Chicken breasts, Food preservation, γ-irradiation, Oxyhalogen disinfectant, Salmide, Sodium chlorite, Shelf-life extension

## Abstract

A novel portable chamber was developed to extend the shelf life of chicken breasts through a synergistic treatment of gamma irradiation and Salmide®, a sodium chlorite-based oxy-halogen. This combination successfully enhanced the shelf life by utilizing a low dosage of gamma irradiation alongside low concentrations of Salmide (200 ppm sodium chlorite). Fresh chicken breast samples were treated with gamma irradiation, then packed in ice containing Salmide within the portable chamber, and subsequently stored for 20 days in a refrigerator at 4 °C ± 1. The study investigated aerobic bacterial counts, sensory analysis, and Thiobarbituric acid (TBA) levels. Results showed that Salmide alone significantly reduced microbial counts and extended shelf life by 8 days. Gamma irradiation at 1 kGy, either alone or combined with Salmide, caused a sequential reduction in total aerobic bacterial counts by 2,3 logarithmic cycles, respectively, extending the storage period to 12 days. Furthermore, a 16 day shelf life extension was achieved with gamma irradiation at 3 kGy, either alone or in combination with Salmide, resulting in a reduction of total aerobic bacteria by 5 logarithmic cycles. This study is the first to employ Salmide in conjunction with gamma irradiation as an innovative technology in a portable chamber to enhance the safety and shelf life of chicken breasts during storage in the designed portable chamber.

## Introduction

For thousands of years, Egyptians have preserved fresh foods, including poultry, through methods such as drying with salt, bandaging, and covering bundles with resins (Ikram 2008; Bhat et al. 2012; Joardder & Masud 2019). Extending the shelf life of foods while maintaining safety and quality is a critical issue for the food industry. The practice of food preservation dates back to prehistoric times and has evolved into various methods, including drying, refrigeration, fermentation, freezing, irradiation, and the addition of chemicals (Joardder & Masud 2019; Dincer 2023). The shelf life of food refers to the duration during which its quality remains acceptable under different conditions of distribution, storage, and display. Spoilage is the process by which food deteriorates, rendering it unacceptable for human consumption or reducing its quality to an inappropriate level for sale (Tarlak 2023). In poultry, spoilage can be detected through the analysis of nitrogenous components that produce off-flavor volatile compounds (aldehydes, ketones, and esters) at the point of spoilage (Alasalvar et al. 2011). There is a pressing need for new preservation methods to reduce food waste by extending the shelf life of perishable foods, particularly in developing countries where food waste poses significant challenges. According to statistics from the State Information Service (SIS) in April 2022, Egypt's total poultry production volume is about 1.4 billion birds, with investments in the poultry industry reaching approximately LE100 billion. However, the Egyptian poultry industry faces numerous challenges (SIS 2022). Contamination of chicken meat products can occur during initial processing, packaging, and storage, leading to potential exposure to pathogenic bacteria such as *E. coli* (Parvin et al., 2020), *Salmonella spp*., *Campylobacter* (Thames & Sukumaran 2020), and *Staphylococcus aureus* (Ahmed et al. 2023). Coliform bacteria are often associated with inadequate sanitary conditions (Bai et al. 2022). Chicken meat is particularly susceptible to pathogenic and spoilage microorganisms, including lactic acid bacteria, *Salmonella spp., Escherichia coli*, *Campylobacter spp*., *Staphylococcus aureus*, and *Pseudomonas spp*. Spoilage is influenced by intrinsic factors such as pH, water availability, and nutrient levels, as well as extrinsic factors like transportation, processing, and storage conditions (Rouger et al. 2017). Common methods to inhibit bacterial growth in chicken include applying heat, dehydrating the meat, and adding preservatives. However, these techniques may alter the meat’s chemical and physical properties (Gómez et al. 2020). Processing methods that minimally affect food quality have become increasingly important to meet consumer demand for minimally processed products. The application of irradiation makes food safer for consumers (FDA 2022). Radiation disrupts the DNA of microbes and other cellular components, rendering them incapable of reproduction and ultimately causing their death (Borrego-Soto et al. 2015). However, high-dose irradiation can lead to undesirable changes in the physical, chemical, and sensory characteristics of poultry meat (Baptista et al. 2014).

In this manuscript, we present a novel portable storage chamber designed to preserve chicken breasts using Salmide and low doses of gamma irradiation, stored in a refrigerator at 4 °C. Salmide is a sodium chlorite-based oxyhalogen disinfectant (Mullerat et al. 1994) and is utilized as an antimicrobial agent in current poultry industry practices (CFR 2024). This study aims to explore how these modern techniques can synergistically combat microbial proliferation, extend shelf life, and maintain the quality of chicken breast samples through Hurdle technology. A comprehensive investigation, including microbiological, sensory, and physicochemical analyses, was conducted to evaluate the efficacy of these preservation methods over a 20 day storage period at 4 °C ± 1. Gamma irradiation is a proven method for prolonging the shelf life of food products while ensuring safety through the reduction of microbial contamination. The oxidative properties of Salmide®, in combination with the penetrating power of gamma irradiation, provide enhanced microbial control, offering greater efficacy than either method alone. Our findings present a novel alternative storage unit for both the scientific community and the food industry, enhancing food safety and quality while minimizing waste. This unit can also benefit ordinary consumers by saving money, ensuring safety and quality, and meeting the increasing demand for high-quality, durable food products.

## Materials and methods

### Chicken breast samples

**Fig. 1:**
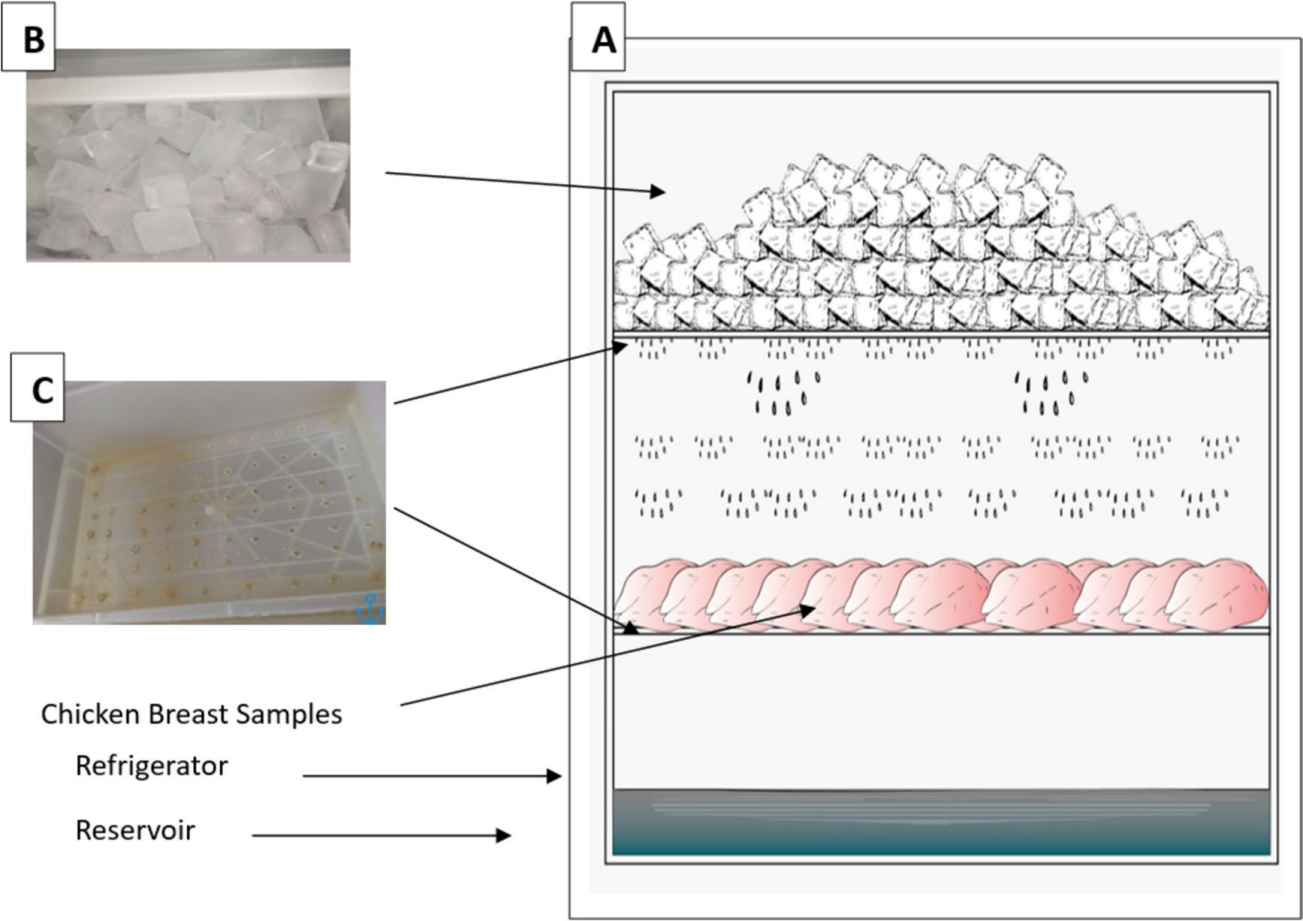
Schematic diagram of the portable chamber. Chicken breast samples were stored on the middle shelf under the chopped ice cubes (4 × 2 × 3 cm) on the top shelf as shown above “B “. Both the top and middle shelves contained enough holes “C” to allow free drainage of melted ice, and then the drainage water were collected in the bottom shelf “the reservoir”

Fresh chicken breast samples were purchased from a local poultry shop in Cairo, Egypt. The samples were subjected to the proposed treatment and stored in the portable storage cabinet for 20 days at 4 °C ± 1, as illustrated in Fig. 1. Control and test samples were aseptically obtained by cutting 20 g from each chicken breast using sterile scissors and tweezers. Microbiological, sensory, and physiochemical analyses were conducted every 4 days.

### Preparation of frozen salmide ice

Frozen salmide ice was prepared by mixing 26 ml of Salmide (200 ppm sodium chlorite) into 20 L of sterilized tap water. The solution was poured into four plastic ice molds and frozen at − 20 °C for three days. The resulting ice cubes (4 × 2 × 3 cm) were stored in the freezer and added to the storage chamber as needed. Notably, the ice cubes gradually melted during the storage period in the refrigerator at 4 °C ± 1, creating a saline solution that dripped onto the surface of the chicken breasts in the storage chamber (Fig. 1).

## Shelf-life extension of chicken breasts using the portable storage chamber

### Use of portable storage chamber with salmide treatment

Samples were immediately submerged in 2 L of tap water (20 °C) containing 200 ppm sodium chlorite for 1 min and then drained for 1 min. The tap water with sodium chlorite was used exclusively to wash the samples and reduce contamination levels before storage, rather than as a secondary treatment. Thus, the samples were not exposed to sodium chlorite more than once during this process. The samples were then stored on the middle shelf of the portable chamber, positioned beneath the top shelf containing the ice cubes. Both shelves had sufficient drainage holes to allow melted ice to flow freely, keeping the surface of the chicken breasts wet at all times, with the melted ice collected in a reservoir at the bottom (Fig. 1). The portable chamber was maintained in the refrigerator at 4 °C, with ice cubes replenished and the reservoir emptied every 3 days. Control samples were stored on another shelf at 4 °C in the refrigerator.

### Use of portable storage chamber with synergism of gamma irradiation and salmide

Gamma irradiation of the chicken breast samples was conducted using a Cobalt-60 (60Co) Gamma Cell GC 220, produced by Canada Co. Ltd. and located at the National Center for Radiation and Technology (NCRRT) in Nasr City, Cairo, Egypt. The samples were exposed to different dosages of gamma irradiation (1 and 3 kGy) at ambient temperature and were not submerged in tap water containing 200 ppm sodium chlorite. The dose rate of the irradiator at the time of exposure was 0.829 kGy/h.

Dosimetry was performed using a 5 mm diameter alanine dosimeter (Bruker Instruments, Rheinstetten, Germany), calibrated according to international standards to ensure accurate dose measurement. The actual doses measured were within 3% of the target doses, which is typical for irradiation procedures. The Dose Uniformity Ratio (DUR) was 1.2, based on previous calibration conducted by our dosimetry lab using a reference alanine pellet dosimetry system. The samples measured 3 × 3 cm, which may affect the irradiation dose distribution. Irradiated samples were divided into two groups: control samples were kept in the refrigerator, while tested irradiated samples were stored on the middle shelf of the storage cabinet, beneath the melted salmide ice from the top shelf.

## Microbiological analysis

Total aerobic plate counts (APCs), coliform, and fecal coliform (*Escherichia coli*) counts were investigated every 4 days (0, 4, 8, 12, 16, 20 days) during the storage period, following standard methods cited by APHA (1992). Total aerobic bacterial counts were determined using the dilution plate method with plate count agar, incubated at 37 °C for 24 h. Each value represents the mean of three samples and is expressed logarithm base colony-forming units per gram (log cfu/g). Total coliform bacteria were assessed using MacConkey broth via the Most Probable Number (MPN) technique with three tubes, as described by WHO (1993). Inoculated tubes were incubated at 37 °C for 24–48 h, with the presence of acid (yellow) and gas (in Durham’s tubes) indicating positive results. *E. coli* was further determined through the MPN technique: positive MacConkey broth tubes were agitated, and a loopful was transferred to new MacConkey broth tubes, which were then incubated at 44.4 °C for 48 h (APHA 1992). Positive cultures were streaked onto Eosin Methylene Blue agar and incubated at 37 °C for 24 h, with positive colonies exhibiting a greenish metallic sheen in reflected light.

## Physiochemical analysis

### pH values

Samples were tested for pH values every 4 days (0, 4, 8, 12, 16, 20 days) during the storage period using a Wincom pH-8414 Portable pH meter with a glass electrode at room temperature (AOAC 2000).

### 2-thiobarbituric acid (TBA) procedure

TBA values of chicken breast samples were determined according to Tarladgis et al. (1960) using a spectrophotometer. Absorbance at 531 nm was measured and multiplied by a factor of 7.8, with TBA values expressed as milligrams of Malondialdehyde (MDA) per kilogram of chicken breast.

### Sensory analysis

A total of 20 g samples were taken every four days, divided into 10 g for total bacterial count and 10 g for sensory analysis, following the guidelines of Meilgaard et al. (2006). A 9-point hedonic scale was used, where 9 = like extremely and 1 = dislike extremely. A score of 4 or below was deemed unacceptable, indicating the end of shelf life. A group of 20 trained panelists evaluated the samples, which were coded and presented in sterilized petri dishes (90 mm). The attributes assessed included odor, color, and overall acceptability. Statistical analyses were performed using SAS software version 9 (SAS 2002).

Data collection focused on the effectiveness of treatments in extending the shelf life of chicken breasts stored at 4 °C, limited to periods before samples were deemed unfit for consumption to ensure reliable results.

## Statistical analysis

All experiments were conducted with three independent replicates, and results are presented as mean ± SD (n = 3) to ensure robust data collection. The experimental design included:

- Control Group: Untreated chicken breasts.

- Treatment Group 1: Chicken breasts treated with Salmide at specified concentrations.

- Treatment Group 2: Chicken breasts subjected to gamma (γ) irradiation at doses of 1 kGy and 3 kGy.

- Combination Treatment: Chicken breasts treated with both Salmide and γ-irradiation.

Data were analyzed using the SAS software package, version 9 (SAS 2002). Statistical significance between treatment groups was assessed using two-way analysis of variance (ANOVA), with post hoc comparisons performed using Duncan’s Multiple Range Test (Duncan 1955). A significance level of P ≤ 0.05 was considered statistically significant.

## Results

### Effect of salmide/gamma irradiation and refrigeration storage on the microbial load of chicken breast

The portable chamber effectively extended the shelf life of chicken breast using Salmide and low doses of gamma irradiation. The effects of gamma irradiation and 200 ppm sodium chlorite on shelf life were systematically investigated over a 20 day storage period at a controlled temperature of 4 °C ± 1. Microbial counts, expressed as logarithm base colony-forming units per gram (log cfu/g), consistently increased during storage. Control samples recorded 6.15 ± 0.04 log cfu/g after 4 days and exhibited sensory deterioration, leading to complete rejection after 8 days with a microbial count of 7.05 ± 0.09 log cfu/g. Salmide-treated chicken breast samples had a microbial count of 6.25 ± 0.08 log cfu/g after 8 days and were rejected after 12 days, with a count of 7.26 ± 0.09 log cfu/g. This indicates the inhibitory effect of Salmide, extending sensory acceptance to 8 days compared to 4 days for control samples.

After 12 days, chicken samples treated with 1 kGy gamma irradiation alone had a microbial count of 6.93 ± 0.04 log cfu/g and were rejected after 16 days with a count of 7.24 ± 0.09 log cfu/g. Conversely, samples treated with 3 kGy yielded a count of 5.31 ± 0.08 log cfu/g after 16 days. When combining 1 kGy irradiation with 200 ppm sodium chlorite, the total microbial count was 5.18 ± 0.08 log cfu/g after 12 days. The combination of 3 kGy and 200 ppm sodium chlorite resulted in a count of 4.69 ± 0.08 log cfu/g after 16 days and was rejected after 20 days with a count of 5.38 ± 0.09 log cfu/g. These findings highlight the efficacy of both irradiation doses and sodium chlorite in reducing the microbial load of chicken breast samples stored at 4 °C (Table 1).

**Table 1:** Total Microbial Counts of Chicken Breast Samples treated with γ-irradiation and salmide during the storage (4 °C ± 1)

Days	Total aerobic mesophilic bacteria (log cfu/g)					
	Treatments					
	Control	Salmide	1.0	1.0 + salmide	3.0	3.0 + salmide
0	5.46^a^_a_ ± 0.08	4.44^b^_a_ ± 0.06	3.73^c^_a_ ± 0.09	2.31^d^_a_ ± 0.05	< 10	< 10
4	6.15^a^_b_ ± 0.04	5.90^b^_b_ ± 0.08	4.13^c^_b_ ± 0.07	3.95^d^_b_ ± 0.08	2.13^e^_a_ ± 0.01	< 10
8	7.05^a^_c_ ± 0.09R	6.25^b^_c_ ± 0.08	5.00^ cc^ ± 0.08	4.71^d^_c_ ± 0.05	3.74^e^_b_ ± 0.05	2.18^f^_a_ ± 0.01
12	R	7.26^a^_d_ ± 0.09R	6.93^b^_d_ ± 0.04	5.18^ cd^ ± 0.08	4.77^d^_c_ ± 0.08	3.53^e^_b_ ± 0.09
16	R	R	7.24^a^_e_ ± 0.09R	6.38^b^_e_ ± 0.06R	5.31^ cd^ ± 0.08_d_	4.69^d^_c_ ± 0.08
20	R	R	R	R	6.05^a^_e_ ± 0.09R	5.38^b^_d_ ± 0.09R
	R	R	R	R	R	R

R = samples sensorially rejected. < 10 = below detectable limit (< 10 cfu/ml). Mean values followed by different superscripts (within rows) and different subscripts (within columns) are significantly different (p < 0.05)

### Effect of salmide/gamma irradiation and refrigeration storage on coliform and *E. coli* contamination in chicken breast

Data from the Most Probable Number (MPN) analysis of chicken breast treated with Salmide showed a dramatic reduction to 2400 MPN/ml after 8 days of storage, compared to > 110,000 MPN/ml in control samples. The MPN of *E. coli* in treated samples was reduced to 460 MPN/ml, while the control recorded 24,000 MPN/ml. This indicates that Salmide significantly extends the shelf life of chicken breast products up to 8 days. When treated with 1 kGy after 12 days at 4 °C, the samples reported an MPN of 2400. Notably, the synergy between 1 kGy gamma irradiation and 200 ppm sodium chlorite completely eliminated coliform and *E. coli* counts to < 3 MPN/ml. Control samples and those treated with Salmide or 1 kGy alone showed growth throughout the storage period. In contrast, samples treated with the combination of Salmide and 1 kGy maintained coliform and *E. coli* levels below detectable limits throughout storage. Furthermore, an MPN of coliform and *E. coli* was significantly reduced to < 3 MPN/ml when samples were treated with 3 kGy and 200 ppm sodium chlorite throughout the entire storage period (Table 2).

**Table 2:** Effect of γ-irradiation and 200 ppm salmide on the coliforms and *Escherichia coli* (MPN/ml) during the subsequent storage (4 °C ± 1) of chicken breasts

Days	a. Most probable number of coliforms (MPN/ml)					
	Treatments					
	Control	Salmide	1.0	1.0 + salmide	3.0	3.0 + salmide
0	2400	1100	23	< 3	< 3	< 3
4	24,000	2300	75	< 3	< 3	< 3
8	> 110000R	2400	92	< 3	< 3	< 3
12	R	9200R	2400	< 3	< 3	< 3
16	R	R	9200R	< 3	< 3	< 3
20	R	R	R	R	< 3	< 3

Days	b. Most probable number of *E.coli* (MPN/ml)					
	Treatments					
	Control	Salmide	1.0	1.0 + salmide	3.0	3.0 + salmide
0	1100	150	< 3	< 3	< 3	< 3
4	2400	240	< 3	< 3	< 3	< 3
8	24000R	460	< 3	< 3	< 3	< 3
12	R	1100R	< 3	< 3	< 3	< 3
16	R	R	< 3	< 3	< 3	< 3
20	R	R	R	R	< 3	< 3

R = Samples sensory rejected. < 3 = No positive tubes have been shown in the first three dilutions

### Effect of salmide/gamma irradiation and refrigeration storage on ph of chicken breasts

The pH of control chicken breasts recorded at 5.82 ± 0.01 indicated an acidic medium. In contrast, treated samples with Salmide and gamma irradiation (1.0 kGy and 3.0 kGy) showed significant increases in pH during storage (Table 3). Control samples had a pH of 6.52 ± 0.01 after 4 days and were rejected after 8 days when the pH reached 7.00 ± 0.01. Chicken breasts treated with Salmide lasted for 8 days and were rejected after 12 days with a pH of 7.01 ± 0.01. Similarly, the application of 1 kGy significantly extended shelf life to 12 days, with rejection occurring after 16 days when the pH reached 7.21 ± 0.01. Both gamma irradiation doses (1 kGy and 3 kGy) and Salmide treatment exhibited comparable storage time extensions, maintaining good quality up to 12 days, with rejection after 16 days and pH values reaching 7.19 ± 0.005.

**Table 3:** pH variation of chicken breasts of γ-irradiation and salmide treatments during subsequent storage at 4 °C ± 1

Days	pH values					
	Treatments					
	Control	Salmide	1.0	1.0 + salmide	3.0	3.0 + salmide
0	5.82^a^_a_ ± 0.01	5.81^a^_a_ ± 0.005	5.80^a^_a_ ± 0.005	5.80^a^_a_ ± 0.005	5.80^a^_a_ ± 0.01	5.80^a^_a_ ± 0.01
4	6.52^a^_b_ ± 0.01	6.05^b^_b_ ± 0.005	6.04^b^_b_ ± 0.01	6.04^b^_b_ ± 0.005	6.02^b^_b_ ± 0.005	6.02^b^_b_ ± 0.01
8	7.00^a^_c_ ± 0.01R	6.50^b^_c_ ± 0.005	6.78_c_^c^ ± 0.005	6.77^c^_c_ ± 0.01	6.12^d^_c_ ± 0.01	6.12^d^_c_ ± 0.005
12	R	7.01^a^_d_ ± 0.01	6.85^b^_d_ ± 0.01	6.80^b^_d_ ± 0.01	6.52^c^_d_ ± 0.01	6.50^c^_d_ ± 0.005
16	R	R	7.21^a^_e_ ± 0.01	7.19^a^_e_ ± 0.005	6.80^b^_e_ ± 0.01	6.79^b^_e_ ± 0.01
20	R	R	R	R	7.21^a^_f_ ± 0.01	7.20^a^_f_ ± 0.01
	R	R	R	R	R	R

R = Samples sensory rejected, Mean values followed by different superscripts (within rows) and different subscripts (within columns) are significantly different (p < 0.05)

When 3 kGy was applied alone, pH values reached 6.80 ± 0.01 after 16 days. Impressively, combining 3 kGy with Salmide treatment extended storage time to 16 days, underscoring the synergistic effects of higher irradiation doses and Salmide treatment in enhancing shelf life. Overall, results indicated considerable improvements in storage time with both irradiation doses, particularly with the 3 kGy plus Salmide treatment, which maintained acceptable pH values for up to 16 days (Table 3).

### Effect of salmide/gamma irradiation and refrigeration storage on sensory scores of chicken breast

**Fig. 2:**
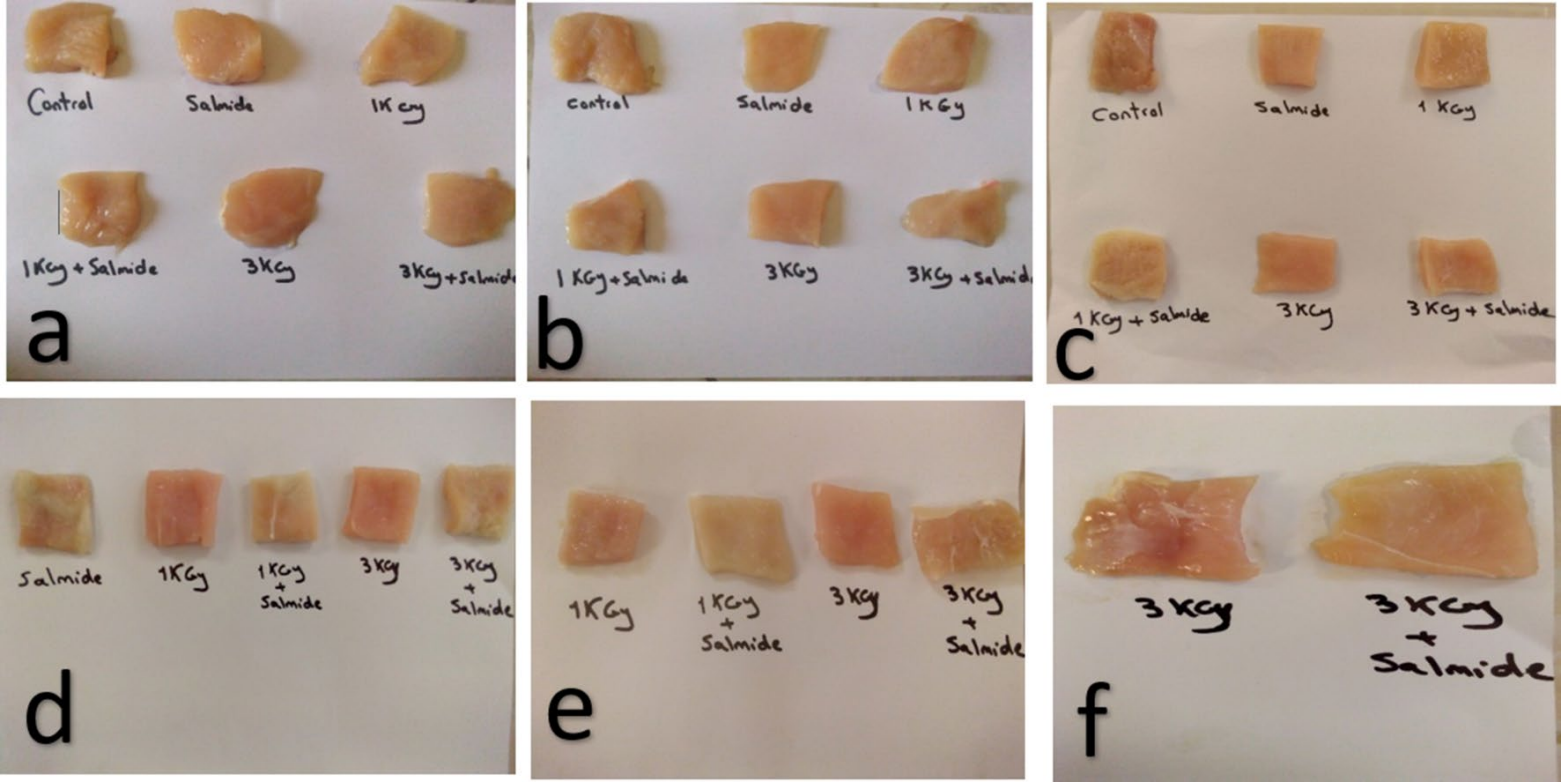
Sensory analysis of chicken breasts treated with different dosage of Gamma irradiation and/or Salmide during storage periods at 4 °C ± 1 as follows: **a** sensory analysis at zero time, **b** sensory analysis after 4 days, **c** sensory analysis after 8 days, **d** sensory analysis after 12 days, **e** sensory analysis after 16 days, **f** sensory analysis after 20 days of storage

Sensory evaluation for color, odor, and overall acceptability was conducted using a 9-point hedonic scale (Table 4). Throughout refrigeration at 4 °C ± 1, sensory characteristics of all chicken breast samples significantly decreased. Control samples were rejected after 8 days due to off-odors and poor taste, while visible spoilage was noted after 4 days. Chicken samples treated with Salmide were rejected after 12 days. Samples treated with 1.0 kGy alone or in combination with Salmide were rejected after 16 days. Chicken breasts exposed to 3.0 kGy, either alone or with Salmide, extended shelf life to 16 days and were rejected after 20 days. This underscores the synergistic effect of higher irradiation doses and Salmide treatment in enhancing shelf life, as illustrated in Fig. 2 and Table 4. Overall, both irradiation doses (1 kGy and 3 kGy) in conjunction with Salmide significantly improved sensory analysis during storage, with the 3 kGy plus Salmide treatment maintaining acceptable sensory quality for up to 16 days (Table 4).

**Table 4:** Effect of γ-irradiation and salmide (200 ppm) treatments on the sensory scores of chicken breasts during subsequent storage (4 °C ± 1)

Days	Control	Salmide	1.0	1.0 + salmide	3.0	3.0 + salmide
	Treatments					
a. Sensorv oder scores						
0	8.9^a^_a_ ± 0.05	8.9^a^_a_ ± 0.05	8.8^a^_a_ ± 0.05	8.8^a^_a_ ± 0.10	8.7^a^_a_ ± 0.05	8.7^a^_a_ ± 0.05
4	6.7^a^_b_ ± 0.05	8.6^b^_a_ ± 0.05	8.7^b^_a_ ± 0.25	8.7^b^_a_ ± 0.05	8.6^b^_a_ ± 0.05	8.6^b^_a_ ± 0.05
8	3.7^a^_c_ ± 0.25R	6.5^b^_b_ ± 0.10	7.8^c^_b_ ± 0.10	7.7^c^_b_ ± 0.05	8.3^d^_b_ ± 0.05	8.4^d^_b_ ± 0.05
12	R	3.8^a^_c_ ± 0.05R	6.0^b^_c_ ± 0.25	6.1^b^_c_ ± 0.25	7.5^c^_c_ ± 0.10	7.7^c^_c_ ± 0.10
16	R	R	3.5^a^_d_ ± 0.05R	3.6^a^_d_ ± 0.15R	6.1^b^_d_ ± 0.25	6.0^b^_d_ ± 0.05
20	R	R	R	R	3.4^a^_e_ ± 0.05R	3.5^a^_e_ ± 0.25R
b. Sensorv color scores						
0	8.9^a^_a_ ± 0.05	8.8^a^_a_ ± 0.05	8.8^a^_a_ ± 0.05	8.7^a^_a_ ± 0.05	8.8^a^_a_ ± 0.05	8.7^a^_a_ ± 0.05
4	8.5^a^_b_ ± 0.05	8.0^a^_b_ ± 0.10	8.2^a^_b_ ± 0.10	8.0^a^_b_ ± 0.05	8.1^a^_b_ ± 0.10	8.0^a^_b_ ± 0.25
8	6.6^a^_c_ ± 0.25R	7.1^b^_c_ ± 0.15	7.8^c^_c_ ± 0.05	6.2^d^_c_ ± 0.25	7.5^e^_c_ ± 0.05	6.8^f^_c_ ± 0.10
12	R	5.0^a^_d_ ± 0.05R	6.7^b^_d_ ± 0.05	5.0^c^_d_ ± 0.25	7.0^d^_d_ ± 0.05	5.5^e^_d_ ± 0.10
16	R	R	5.1^a^_e_ ± 0.25R	3.7^b^_e_ ± 0.05R	6.2^c^_e_ ± 0.15	4.0^d^_e_ ± 0.25
20	R	R	R	R	5.0^a^_f_ ± 0.05R	3.2^b^_f_ ± 0.05R
c. Overall acceptability sensory scores						
0	8.7^a^_a_ ± 0.05	8.6^a^_a_ ± 0.05	8.6^a^_a_ ± 0.05	8.5^a^_a_ ± 0.05	8.6^a^_a_ ± 0.05	8.5^a^_a_ ± 0.05
4	6.6^a^_b_ ± 0.05	8.4^b^_a_ ± 0.10	8.4^b^_a_ ± 0.15	8.4^b^_a_ ± 0.05	8.3^b^_a_ ± 0.15	8.3^b^_a_ ± 0.05
8	3.7^a^_c_ ± 0.25R	7.0^b^_b_ ± 0.15	7.4^c^_b_ ± 0.05	7.5^c^_b_ ± 0.25	7.8^d^_b_ ± 0.05	7.9^d^_b_ ± 0.10
12	R	3.7^a^_c_ ± 0.05R	6.0^b^_c_ ± 0.05	6.2^b^_c_ ± 0.25	6.9^c^_c_ ± 0.05	7.0^ cc^ ± 0.10
16	R	R	3.3^a^_d_ ± 0.25R	3.5^a^_d_ ± 0.05R	6.6^b^_d_ ± 0.15	6.7^b^_d_ ± 0.25
20	R	R	R	R	3.6^a^_e_ ± 0.05R	3.9^a^_e_ ± 0.05R

Where 0 ( dislike extremely) to 9 ( like extremely)

R = samples sensorially rejected

Mean values followed by different superscripts (within rows) and different subscripts (within columns) are significantly different (p < 0.05)

### Effect of salmide/gamma irradiation and refrigeration storage on thiobarbituric acid reacting substances of chicken breast

Results indicated that lipid peroxidation increased throughout storage, with a significant rise (p ≤ 0.05) in TBA values. The TBA value reached 1.06 mg/kg on day 8, coinciding with microbiological and sensory rejection. Samples treated with Salmide were rejected on day 12, showing a TBA value of 0.95 mg/kg, with no significant increases prior.

Notably, gamma irradiation (1 kGy) alone, as well as in combination with Salmide, extended shelf life, maintaining acceptability until day 12. However, samples were rejected after 16 days when TBA levels reached 0.95 mg/kg and 1.20 mg/kg, respectively. Samples treated with both gamma irradiation (3 kGy) and the combination of 3.0 kGy plus Salmide maintained shelf life up to 16 days, with rejection after 20 days when TBA values reached 2.19 mg/kg and 2.86 mg/kg, respectively (Table 5).

**Table 5:** TBA value of chicken breasts of γ-irradiation and salmide treatments and subsequent storage (4 °C ± 1)

Days	TBA value ( mg MDA/ kg)					
	Treatments					
	0.0	Salmide	1.0	1.0 + salmide	3.0	3.0 + salmide
0	0.65^a^_a_ ± 0.03	0.67^b^_a_ ± 0.005	0.73^c^_a_ ± 0.02	0.90^d^_a_ ± 0.02	0.95^e^_a_ ± 0.08	1.00^f^_a_ ± 0.01
4	0.75^a^_b_ ± 0.02	0.70^b^_b_ ± 0.01	0.76^c^_b_ ± 0.01	0.92^d^_b_ ± 0.01	1.00^e^_b_ ± 0.005	1.06^f^_b_ ± 0.02
8	1.06^a^_c_ ± 0.03R	0.74^b^_c_ ± 0.01	0.82_ cc_ ± 0.005	0.96^d^_c_ ± 0.01	1.02^e^_c_ ± 0.02	1.09^f^_c_ ± 0.04
12	R	0.95^a^_d_ ± 0.04R	0.88^b^_d_ ± 0.01	1.03^ cd^ ± 0.03	1.08^d^_d_ ± 0.04	1.14^e^_d_ ± 0.03
16	R	R	0.95^a^_e_ ± 0.09R	1.20^b^_e_ ± 0.02R	1.95^c^_e_ ± 0.03	2.18^d^_e_ ± 0.04
20	R	R	R	R	2.19^a^_f_ ± 0.01R	2.86^b^_f_ ± 0.07R

R = samples sensorially rejected

Mean values followed by different superscripts (within rows) and different subscripts (within columns) are significantly different (p < 0.05)

## Discussion

This study demonstrates the efficacy of an innovative preservation approach using a novel portable storage cabinet that combines Salmide—a sodium chlorite-based oxyhalogen disinfectant—with gamma irradiation to extend the shelf life of chicken breasts. The design of this portable chamber allows for easy use in the food industry, by individual consumers, and in retail stores. The water reservoir at the chamber's bottom collects drainage from the first and second compartments, which is then discarded, preventing any contact with the chicken breasts. This design ensures that sensory properties such as texture and color are preserved, eliminating the need for further analysis of these parameters in this context. Our findings are consistent with recent studies investigating the antimicrobial effects of irradiation and antimicrobial compounds in food preservation (Li et al. 2022; Shankar et al. 2022).

Microbiological analysis revealed a consistent reduction in microbial counts with Salmide treatment, emphasizing its antimicrobial properties. Salmide effectively extended the sensory acceptance period, delaying rejection until Day 12. Similar results were observed with trisodium phosphate, which extended poultry shelf life to 12 days while inhibiting aerobic plate counts (Kim and Marshall 1999). Additionally, acidified sodium chlorite has been shown to reduce microbial contamination on whole chicken carcasses, with both 450 and 900 ppm concentrations significantly decreasing total viable bacteria, *Campylobacter, and Salmonella* counts (McWhorter et al. 2022).

The acceptable doses of gamma irradiation are outlined in the Code of Federal Regulations (CFR). For instance, a maximum dose of 4.5 kGy is permitted for refrigerated meat products, while 7.0 kGy is set for frozen products (21 CFR 179.26). Gamma irradiation is recognized as a low-temperature preservation technology that effectively reduces microbial loads and extends the shelf life of fresh meat, enhancing food safety and quality by destroying pathogens and inhibiting spoilage microorganisms (Zhang et al. 2020). In our study, we successfully administered doses of 1 kGy and 3 kGy, which fall within the upper limits established by the CFR. Our results indicate that these doses significantly reduced the total aerobic bacterial count by 2 and 5 logarithmic cycles, respectively, demonstrating the effectiveness of irradiation in decreasing the growth of microorganisms in chicken breast samples.

Comparing our findings with recent literature, we align with Huang et al. (2023), who reported that irradiation significantly decreased microbial counts and positively impacted the overall quality of smoked chicken breast. Likewise, Li et al. (2022) noted that irradiation improved the microbial quality of frozen duck meat samples. Thus, our findings support the efficacy of gamma irradiation in maintaining food safety while simultaneously increasing the shelf life of chicken breasts, reinforcing the core premise of our manuscript.

The combination of gamma irradiation doses (1 kGy and 3 kGy) with Salmide further amplifies shelf life extension, reaching up to 20 days. This synergistic effect aligns with previous studies examining the individual efficacy of Salmide and gamma irradiation in microbial load reduction (Joardder & Masud 2019). The APC count method revealed that the combination of gamma irradiation doses with Salmide resulted in lower log counts (cfu/g) compared to treatments applied alone during storage. This method also provides insights into the keeping quality of poultry carcasses, as reported by El Sayed et al. (2020).

Our research yields better results than other synergistic approaches, such as applying both cinnamon essential oil nanoemulsions and refrigeration to chicken breast fillets, which extended shelf life only to 15 days (Wang et al. 2021). Similar results were seen when low-dose gamma irradiation (2.5 kGy) combined with a chitosan edible coating (2%) containing 0.1% grape seed extract inhibited microbial load and enhanced the sensory quality of chicken breast meat over 21 days of storage at 4 °C (Hassanzadeh et al. 2017). The reduction in coliform and *E. coli* concentrations corroborates the inhibitory effect of Salmide, contributing to a preservation duration of 8 days. The combination of Salmide with irradiation doses of 1 kGy manifests a dual effect, significantly reducing coliform and *E. coli* counts while extending shelf life to 12 days. Notably, the combination of gamma irradiation (3 kGy) and Salmide achieves the most substantial reduction in microbial counts, extending shelf life to 16 days, with rejection occurring after 20 days of storage. This synergistic outcome underscores the potential of these combined treatments to address microbial contamination throughout the storage period.

Sensory evaluations align with microbial findings, confirming the extension of storage time with Salmide and gamma irradiation, whether used alone or in combination. The rejection of control samples after 8 days and visible deterioration after 4 days emphasizes the need for effective preservation methods to maintain product quality. Salmide's ability to delay sensory rejection until Day 12 indicates its effectiveness in mitigating the impact of microbial proliferation on sensory attributes. This finding is consistent with Arshad et al. (2019), where increased gamma irradiation doses led to a decrease in contamination levels for both total bacteria and coliforms. No contamination was documented in the group treated with 2 kGy and turmeric powder, which maintained both aerobic and vacuum packaging.

Our results also agree with Montiel et al. (2013), who noted that irradiation at doses of 1 and 2 kGy significantly reduced total viable counts in smoked salmon by 2 and 2.4 log units, respectively. An et al. (2017) reported that irradiation reduced bacterial loads and improved shelf life, which aligns with our findings. Additionally, another study demonstrated that combining Salmide and EDTA significantly enhances the shelf life of broiler drumsticks, extending freshness and safety until Day 8, surpassing typical shelf life (Mullerat et al. 1994). Notably, our results show even greater preservation, maintaining chicken breast quality for up to 20 days.

Furthermore, our findings indicate significant inhibition compared to cinnamon essential oil, which only improved sensory quality for up to 15 days (Wang et al. 2021). We combined Salmide and gamma irradiation for several reasons, it’s broad-spectrum antimicrobial activity where Salmide has demonstrated wide-ranging effectiveness against bacteria and molds with minimal chemical residues compared to other treatments. Widespread use and stability, Salmide is utilized by various manufacturers to ensure food safety and minimize waste due to its effectiveness and stability under various conditions. Synergy with other preservatives, Salmide exhibits strong synergistic effects when combined with other preservatives, enhancing overall effectiveness while ensuring safe and wholesome products for consumers. Advantages over other chemicals, Many chemical preservatives have notable drawbacks, such as lack of stability, risk of product quality deterioration, high reactivity, potential health risks to users, and higher costs (Mullerat et al. 1994; Abolmaaty et al. 2023).

These factors collectively demonstrate the potential of combining Salmide and gamma irradiation as an effective preservation strategy for extending the shelf life of chicken breasts while maintaining safety and quality.

Salmide® is described in a U.S. patent as "a series of synergistic combinations of chlorine-containing materials that act as microbiocides, virucides, and sporocides without utilizing chlorine dioxide itself" (Gordon, 1989a). Its unique chemical formulation leads to an oscillating chemical reaction, with chlorite, chloride, and chlorate ions as the predominant species. Smaller amounts of superoxide and hypochlorite ions, along with reactive intermediates (e.g., Cl₂O₂, HCl₂O₂, Cl₂O₄) and trace amounts of chlorine dioxide, are also generated (Gordon, 1989b). When Salmide® interacts with microorganisms, organic matter, or transition metals, or becomes acidified, this oscillating equilibrium shifts, converting stable chlorite and chlorate ions into more reactive species that enhance microbial killing. Given the controlled and minimal formation of potentially harmful byproducts, we did not measure these components, as they exist in very small, non-toxic amounts under the storage conditions used. This distinction clarifies the difference between washing and treating, providing a detailed explanation of Salmide®'s chemical behavior while addressing concerns regarding toxic components.

To manage the odor of sodium chlorite in samples, we implemented several key steps: First Controlled Concentration, We meticulously controlled the concentration of sodium chlorite used, adhering to guidelines from Gordon's 1989 patent. Second Low Concentration,we utilized a low parts per million (ppm) concentration that falls below the acceptable levels specified in the CFR—specifically, the Code of Federal Regulations Title 21 (21 CFR 173.325), which permits sodium chlorite as an antimicrobial agent in red meat processing at concentrations between 500 and 1,200 ppm. Third Resting Period, After treatment, we allowed the samples to rest for a designated period before presenting them to panelists for sensory evaluation. This resting period was essential for dissipating any residual odor associated with sodium chlorite.

These measures were crucial in ensuring that the sensory qualities of the chicken breasts were preserved while still benefiting from the antimicrobial properties of sodium chlorite.

Lipid peroxidation values highlight the impact of preservation methods on the oxidative stability of chicken breasts. The rejection of control and Salmide-treated samples after 12 days indicates the progression of lipid peroxidation, which may contribute to off-flavors. Gamma irradiation (1 kGy), alone or in combination with Salmide, extends the preservation of lipid quality until Day 16, while the combination of gamma irradiation (3 kGy) and Salmide achieves the most significant extension, reaching 20 days before rejection.

The observed trends in lipid peroxidation suggest that the combined treatment effectively retards oxidative processes, as indicated by a slower rate of TBA increase compared to other treatments, thereby preserving the lipid quality of chicken breasts over an extended storage period. Acceptable levels for non-rancid meat are typically between 0.02 and 2.55 mg MDA/kg, according to Turcu et al. (2020). This preservation is crucial for maintaining sensory attributes and overall product quality, contributing to consumer satisfaction.

Our lipid peroxidation results contrast with those observed by Abdel-Khalek et al. (2022), who found that polylactic acid pouches containing a mixture of lemongrass and cumin essential oils, combined with 2 kGy gamma irradiation, showed the lowest increase in TBA during the storage of poultry breast meats. In our study, total microbial counts of chicken breast reaching approximately 5.5 log cfu/g were deemed unacceptable by the panelists, leading to sample rejection. This finding underscores the critical importance of microbial quality in chicken products, as significant increases in microbial loads can indicate spoilage and pose food safety risks.

Maintaining microbial counts below this threshold is essential for ensuring the sensory quality of chicken breasts, aligning with existing food safety standards. Elzamzamy (2014) noted that aerobic microflora serves as a standard for predicting the shelf life of foods, with the International Commission on Microbiological Specifications for Foods (ICMSF) recommending a maximum count limit of 7 log cfu/g for mesophilic aerobic bacteria (ICMSF 1986).

Both microbial growth and lipid peroxidation contribute significantly to the development of off-flavors in chicken breasts. As microbial counts rise, various metabolites, including volatile compounds and enzymes, are produced, resulting in undesirable flavors and aromas (Zahid et al. 2018). This dual impact of lipid peroxidation and microbial activity highlights the need for effective control measures in food processing and storage to maintain the sensory qualities and safety of poultry products.

The synergy observed in microbial reduction and shelf-life extension with gamma irradiation doses (3 kGy) and Salmide emphasizes the potential of these combined treatments to meet the stringent safety and quality requirements of the food industry.

In this study, we present an innovative preservation approach utilizing a novel portable storage cabinet that combines Salmide, a sodium chlorite-based oxyhalogen disinfectant, with gamma (γ) irradiation to extend the shelf life of chicken breasts. To our knowledge, this is the first published unit that enables both consumers and business owners to safely store chicken breasts for longer periods, with applicability to other food products as well. This research introduces a unique method that synergistically integrates Salmide and γ-irradiation, demonstrating their combined effectiveness in microbial control and shelf-life extension.

The specially designed portable storage chamber offers practical benefits, making it accessible for use across various settings, including the food industry, individual consumers, and retail stores. This versatility enhances food safety and reduces waste in different sectors. By developing a solution that is user-friendly, we aim to empower consumers and retailers to implement effective preservation techniques that ensure the quality and safety of chicken products. This approach may significantly reduce food waste by preventing spoilage, thereby contributing to food security by minimizing the amount of food discarded.

Our findings underscore the potential of this innovative approach as a significant advancement in food preservation technology, contributing to ongoing efforts to enhance food safety and quality in the industry.

## Conclusion and future directions

The combined application of Salmide and γ-irradiation presents significant potential for extending the shelf life of chicken breasts while maintaining safety and quality attributes. The observed synergistic effects on microbial reduction, sensory attributes, and lipid peroxidation warrant further exploration of these preservation methods. Future research could focus on optimizing the combination ratios of Salmide and γ-irradiation doses, exploring variations in storage conditions, and assessing the economic feasibility of implementing these strategies at an industrial scale.

Additionally, investigating the integration of Gamma irradiation as a complementary technology may further minimize processing effects. This study provides a valuable contribution to ongoing efforts in the food industry to enhance food safety, reduce waste, and meet the evolving consumer preferences for minimally processed, high-quality products. The results offer a strong foundation for future research and practical applications in the field of food preservation.

The lowest irradiation dose used (1 kGy) in combination with 200 ppm sodium chlorite was sufficient to ensure the microbiological safety of chicken breasts, effectively eliminating coliform bacteria and *E. coli* contamination. An irradiation dose of 3.0 kGy, either alone or in combination with 200 ppm sodium chlorite, effectively controlled the microbial flora of chicken breasts, resulting in the complete elimination of coliforms and *E. coli* while maintaining acceptable sensory analysis and Thiobarbituric acid (TBA) values. This combination extended the shelf life to 16 days at refrigeration temperatures, compared to only 4 days for control samples, while remaining within acceptable limits for non-rancid meat.

## Data Availability

No datasets were generated or analysed during the current study.

## Abbreviations

AOAC: Association of official analytical chemists
APHP: American public health association
APCs: Aerobic plate counts
Cfu: Colony forming units
CFR: Code of federal regulations
60 C0: Cobalt 60
DNA: Deoxyribonucleic acid
*E.coli*: Escherichia coli
ICMSF: International commission on microbiological specifications for foods
kGy: Kilo gray
MDA: Malondialdehyde
MPN: Most probable number
NCRRT: National center for radiation research and technology
SIS: State information service
TBA: Thiobarbituric acid
WHO: World health organization
